# Supplementary material for: CiberAMP: An R Package to Identify Differential mRNA Expression Linked to Somatic Copy Number Variations in Cancer Datasets
Source: Biology (Basel). 2022 Sep 28;11(10):1411. doi: 10.3390/biology11101411 (PMC9598370; doi:10.3390/biology11101411)
Supplement: Supplementary file 1 [file biology-11-01411-s001.zip › 2. Bustelo.SupplInfo.V2.pdf]

Supplemental Information for

CiberAMP: AN *R* PACKAGE TO IDENTIFY DIFFERENTIAL mRNA EXPRESSION LINKED TO SOMATIC COPY NUMBER VARIATIONS IN CANCER DATASETS

by

Rubén Caloto *et al*.

This PDF file includes:

Supplementary Figure S1

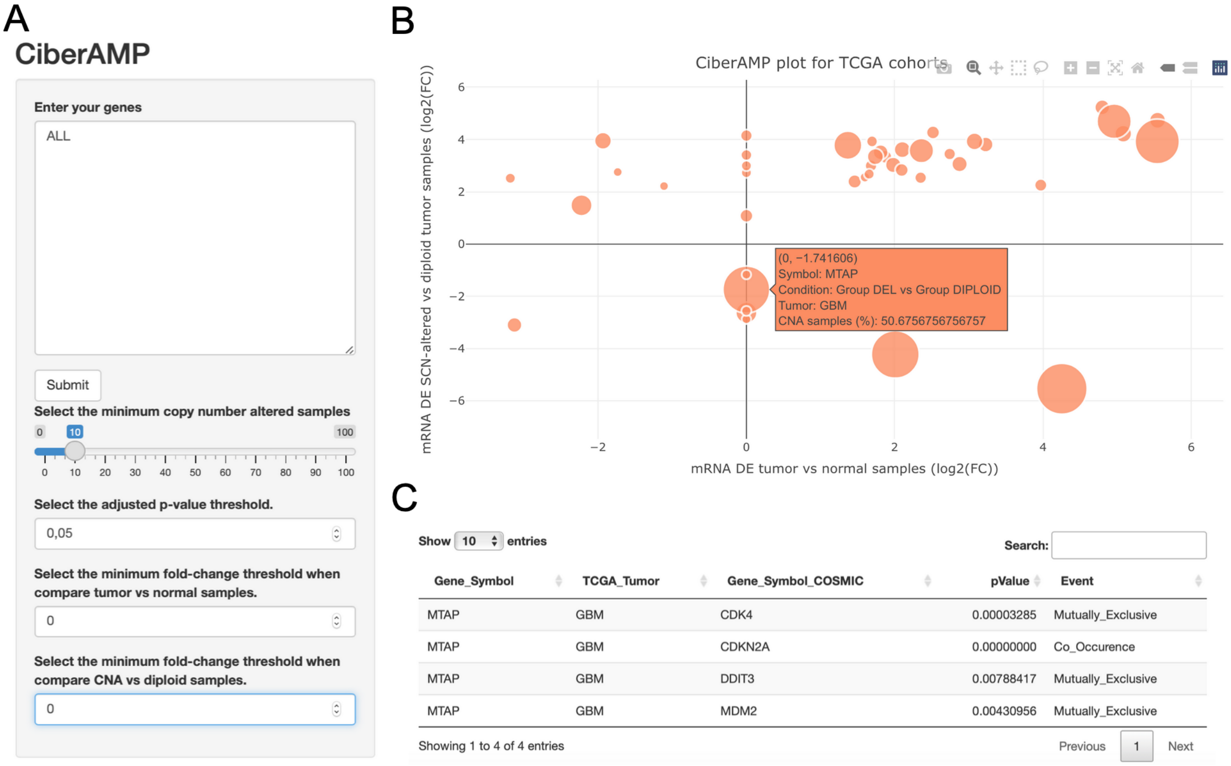

FIGURE S1. Computer screen view of the CiberAMP interface

(A) Area to insert the gene list and the searching parameters by the usuary.

(B) Plot from a CiberAMP analyses showing the identified SCNV-DEGs (brown circles) and the information of one of them. GBM, glioblastoma

(C) Example of the information given by CiberAMP on the cosegregation or mutually exclusivity between the SCNV-DEG selected in A and known proto-oncogenes or tumor suppressor genes.
